# Association of Organ Preservation Methods With Von Willebrand Factor Upregulation in Microvascular Endothelial Cells Posttransplantation: Ex Vivo Lung Perfusion and Static Cold Storage

**DOI:** 10.1097/TXD.0000000000001898

**Published:** 2026-01-12

**Authors:** Parnian Alavi, Sayed Himmat, Nader S. Aboelnazar, Max T. Buchko, Catherine J. Stewart, Keir A. Forgie, Nicholas M. Fialka, Sanaz Hatami, Darren H. Freed, Nadia Jahroudi, Jayan Nagendran

**Affiliations:** 1 Division of Hematology, Department of Medicine, Faculty of Medicine and Dentistry, University of Alberta, Edmonton, AB, Canada.; 2 Division of Cardiac Surgery, Department of Surgery, Faculty of Medicine and Dentistry, University of Alberta, Edmonton, AB, Canada.

## Abstract

**Background.:**

Von Willebrand factor (VWF) is a procoagulant glycoprotein expressed exclusively in endothelial cells and megakaryocytes. It mediates platelet adhesion to endothelial/subendothelial surfaces and initiates thrombogenesis. External stimuli, including hypoxia, were shown to upregulate VWF expression levels and alter its vascular tree expression pattern in the lung. Increased VWF levels are a significant risk factor for thrombus formation, a major complication in organ transplantation. Given that donor organs experience hypoxic conditions during transplantation, this study investigates whether hypoxia alters VWF expression and whether modifying organ preservation to reduce hypoxic exposure could prevent these alterations.

**Methods.:**

Porcine procured lungs were maintained in either static cold storage (SCS) or ex vivo lung perfusion with warm perfusion. Lung tissue biopsies were obtained immediately after organ procurement, 12 h post–cold storage, or post–warm perfusion. VWF RNA and protein expression levels and patterns were analyzed using reverse transcriptase-polymerase chain reaction, Western blot, and immunofluorescence.

**Results.:**

Immunofluorescent analysis demonstrated that transplanted lungs maintained under SCS, but not ex vivo lung perfusion, exhibited VWF expression in an increasing number of microvascular endothelial cells, whereas warm perfusion led to a significant reduction in VWF mRNA levels, and it also showed a clear trend toward reduced protein expression compared with cold storage.

**Conclusions.:**

Increased VWF expression in microvascular endothelial cells under SCS may contribute to transplant-associated thrombogenicity. Decreasing VWF expression through ex vivo normothermic perfusion could significantly mitigate the risk of thrombogenic complications, a major complication of organ transplantation.

## Graphical abstract

**Figure:**
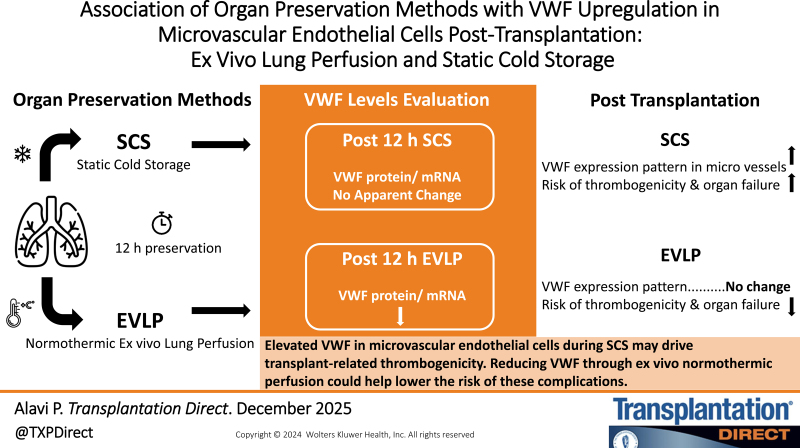

## INTRODUCTION

Organ transplantation, including lung transplant, has become an effective therapeutic option for patients with a variety of end-stage diseases.^1-3^ However, its application is limited because of the shortage of suitable donor organs.^4,5^ Disparity between the supply of transplantable lungs and the demand of potential recipients leads to long waiting times and annual rises in deaths of patients on the lung transplant waiting lists. Consequently, attempts have focused on the use of living donors, marginal donors, and non–heart-beating donors to expand the donor pool.^6^ However, in addition to the problem of supply and demand, patients have to overcome the threat of potential complications from transplantation procedures, including microthrombus formation, which may lead to early allograft failure.

Microthrombus formation is one of the major complications in lung transplantation.^7^ The coagulation process that contributes to vascular thrombosis depends on alterations in the ratio of pro to anticoagulant factors, in favor of the procoagulant molecules.^8,9^ A hypercoagulable state is susceptible to thrombus formation, and a procoagulant molecule, namely von Willebrand factor (VWF), has a major role in this process. VWF is a multimeric, procoagulant glycoprotein that is the primary initiator of thrombus formation. It is produced only in endothelial cells (ECs) and megakaryocytes.^10,11^ In the site of injury, VWF molecules attach to the exposed subendothelial layer and capture platelets to form platelet aggregates, which seal the wall of the damaged vessels.^12-14^ In addition, even in the absence of injury, VWF can be released from ECs in response to some stimuli and attach to the luminal cell surfaces of the intact endothelium.^15^ Under these circumstances, VWF can capture platelets and form platelet aggregates, which may be subsequently stabilized through fibrin deposition, leading to thrombus formation.^16-18^ We have reported that hypoxic conditions, as a stimulus, can upregulate VWF expression.^19,20^ Thus, this hypoxia-driven VWF upregulation may contribute to increased thrombogenicity.^19,20^ Increased levels of VWF have been reported in patients with pulmonary hypertension, which results from a shortage of oxygen in lungs.^21,22^ Organ transplantation is among the conditions that lead to hypoxia exposure of organs as a result of extended disconnection of the donor organs from circulation.^23^ Thus, considering that thrombosis is a major complication of organ transplantation, we hypothesized that hypoxia-induced VWF upregulation may contribute to thrombotic complications during organ transplantation.

We have reported that hypoxic exposure in mice upregulates VWF expression in all major organs, including brain, heart, liver, and lung but not kidney.^19^ Furthermore, specifically in lung but not in the vascular beds of other organs, hypoxia resulted in de novo activation of VWF expression in microvascular ECs, where normally VWF expression is not detected.^19^ We also demonstrated increased platelet adhesion and aggregate formation, as a functional consequence of hypoxia-induced VWF upregulation.^19,20^

One of the most important factors in improving transplantation is an optimal organ preservation protocol to conserve proper function of the donor organ during storage so that the transplanted organ will function optimally after reperfusion.^24,25^ The current standard method for organ preservation is static cold storage (SCS).^26,27^ This method involves cold ischemia, which leads to an extended hypoxic condition due to the lack of oxygen supply to the donor organ for a period of time before transplantation.^23^ However, ex vivo lung perfusion (EVLP) is a novel perfusion-based technique for dynamic lung preservation, which significantly reduces the hypoxia exposure of the donor lung.^28-30^ In this method, the risk of exposure to the cold ischemia and hypoxic condition is minimized by circulating oxygenated, nutrient-rich perfusate to the lung during preservation. The method of organ preservation, particularly the application or absence of lung ventilation, influences the level of hypoxia exposure of donor lungs during transplantation. Therefore, we hypothesized that differential hypoxic exposure could affect VWF expression and the subsequent risk of thrombogenic complications. Although other variables, such as temperature (warm/cold), might have influenced VWF expression and were not examined in this study, the focus on hypoxia was based on our previous research demonstrating that hypoxia is a key factor in VWF upregulation.^19^ In this study, we aim to investigate whether the expression levels of mRNA and protein, as well as the vascular distribution pattern of VWF, are altered in porcine lungs stored in SCS, similar to our observations in hypoxia-treated mice. Additionally, we will examine whether warm perfusion and ventilation during the EVLP procedure influence these effects.

## MATERIALS AND METHODS

All animal experiments in this study were performed under an approved protocol by the University of Alberta Institutional Animal Care Committee. All animal care and use were in accordance with the guidelines of the Canadian Council on Animal Care and the Guide for the Care and Use of Laboratory Animals.

### EVLP Experimental Groups

EVLPs were performed using 2 types of ventilation mode (positive pressure ventilation [PPV] or negative pressure ventilation [NPV]) and the 2 types of perfusate composition materials (acellular [AC] or packed red blood cells [pRBCs]), as previously described.^31^ Twelves porcine lungs from female domestic pigs (42 ± 5 kg) were collected and allocated into 4 different experimental groups with 3 animals per group (AC-PPV, pRBC-PPV, AC-NPV, pRBC-NPV).

### EVLP and SCS Condition

Lungs were perfused under normothermic EVLP for 12 h as described previously.^28^ Briefly, for making the perfusate solution, a freshly standard Krebs-Henseleit buffer (KHB) solution was made for experiments. As a cellular perfusate (RBC), 1.5 L of (KHB) with 8% bovine plus 0.5 L of RBC concentrate were used. The acellular perfusate solution was prepared from 2 L KHB with 8% bovine serum albumin. Under the PPV-EVLP platform, donor lungs were ventilated using a standard intensive care unit ventilator (SERVO-I, Maquet Critical Care AB, Solna, Sweden). On the NPV-EVLP platform, to create a negative pressure inside the sealed organ chamber, a custom-built turbine-driven ventilator was used to produce the normal tidal volume. For the SCS preservation group, the porcine lungs were subjected to an ischemic cold preservation time of 12 h at 4 °C.

### Donor Lung Procurement and EVLP

Animals received a mixture of intramuscular ketamine (20 mg/kg) and atropine sulfate (0.05 mg/kg) to facilitate intratracheal intubation and mechanical ventilation. After that, anesthesia was maintained with isoflurane (1%–3%). A median sternotomy incision exposed the heart, lung, and great vessels. Then, 1000 U/kg of heparin sulfate (Sandoz; QC, Canada) was injected into the right atrial appendage. Next, the animal was exsanguinated, and blood was collected in a cell-saver.^31,32^ The heart was excised first, followed by the lungs. The lungs were carefully dissected from their mediastinal attachments, and then the trachea was clamped at 25 cm H_2_O airway pressure. Lungs were completely excised, and their weight was recorded before ex vivo perfusion started.

According to the experiment protocol, the EVLP circuit was primed with 2 L of the appropriate perfusate. A pulmonary artery (PA) cannula and an appropriate caliber endotracheal tube (ETT) were placed in the PA and the trachea, respectively, and secured with silk ties. The lungs were then transferred to the organ chamber and connected to the EVLP circuit. They were perfused under normothermic conditions for 12 h with a cellular perfusate or a modified Krebs buffer (acellular) and ventilated using either PPV or NPV.^33^ At the end of perfusion, the lungs were weighed, and T12 biopsies were collected from the right lung.

### Left Lung Transplantation

The recipient animal was anesthetized in a similar manner and connected to a mechanical ventilator. Central venous and peripheral arterial lines were inserted for vascular access and telemetry. A clamp-shell incision was performed, and the left lung was clamped off, carefully excised, and discarded. The ventilatory parameters were adjusted to accommodate the single-lung ventilation. Then, the left donor lung was prepared and flushed with 0.5 L of cold (4 °C) low-potassium dextran solution. Next, the left donor lung was implanted into the recipient, and the clamps were removed to ventilate and perfuse the donor graft. After that, the animal was monitored for 4 h. Additionally, lung biopsies were saved from the implanted lung for later analysis.

### Ex Vivo Perfusion of Blood Vessels of Limb

The animals were sedated using ketamine (20 mg/kg), atropine sulfate (0.05 mg/kg), and xylazine (0.9 mg/kg). After orotracheal intubation, general anesthesia was maintained with 1%–3% isoflurane. To prevent clotting, 40 000 IU of heparin was administered during the procedure. The forelimbs were shaved, cleaned, and wrapped in Ioban before procurement. The surgical procedure followed the guidelines outlined by Ozer et al.^34^

### Sample Collection

In vivo (IV) tissue biopsies were obtained immediately upon opening the chest, before exsanguination. Additional tissue biopsies were obtained before SCS or normothermic ex vivo perfusion as (T0) samples. For cold storage conditions, T0 samples were collected immediately after placing the samples in the cooler, representing 0 min after leaving the chest. For the EVLP group, T0 samples were obtained once the lungs were connected to the machine, before the initiation of ventilation or perfusion. Lung tissue biopsies were obtained after 12 h of post–cold storage or post-EVLP as (T12) samples. Biopsies were obtained from the implanted lung after lung transplantation immediately after reperfusion (PT0, posttransplant 0) and after 4 h of reperfusion (PT4). Additionally, vessels of limb were obtained before ex vivo perfusion as (T0) samples and post–warm perfusion as (T12) samples. Collected samples were immediately snap-frozen by immersing them in liquid nitrogen and stored at –80 °C for further analysis. VWF RNA and protein expression levels were analyzed using reverse transcriptase-polymerase chain reaction (RT-PCR) and Western blot.

### VWF Protein Levels Assessment

Tissue biopsies were obtained from the lungs at T0 and T12 in the EVLP or SCS groups. All samples were collected from the peripheral regions of the lungs. Vessels of limb were collected before ex vivo perfusion (T0) and post–warm perfusion (T12). All collected tissue samples were immediately snap-frozen by immersion in liquid nitrogen, stored at –80 °C, and subjected to Western blot analyses as follows.

Protein extraction and determination of protein concentration, and Western blot analyses were performed as previously described.^19^ Briefly, protein samples (25 μg) were separated on 6% sodium dodecyl sulfate polyacrylamide gel and were transferred to a polyvinylidene difluoride membrane. Rabbit anti-VWF and anti-tubulin were used as primary antibodies. Horseradish peroxidase-conjugated goat anti-rabbit antibody was used as the secondary antibody. Tubulin was used as the loading control. Bands were detected and analyzed on a Bio-imaging System.

### Quantitative RT-PCR

Total RNA was isolated from frozen lung tissues and vessels of limb using the RNeasy Mini kit. cDNA was synthesized using the High-Capacity RNA-to-cDNA Master Mix. Quantitative RT-PCR was performed using SYBR Green master mix (Life Technologies) on the ABI 7500 Real-Time PCR System. All PCRs were repeated in triplicate. Bio-Rad PrimePCR SYBR Green Assay VWF and hypoxanthine‑guanine phosphoribosyltransferase primers were used for RT-PCR.

### Immunofluorescence

For immunofluorescent analyses, lung sections were snap-frozen in optimal cutting temperature and sectioned at 5 μm. Immunofluorescent analyses were performed using primary sheep anti-VWF and rabbit anti-CD31 antibodies. Alexa Fluor 568 donkey anti-sheep IgG (H+L) and Alexa Fluor 488 donkey anti-rabbit IgG (H+L) were used as secondary antibodies, respectively. The tissues were incubated with the primary antibody overnight at 4 °C, washed with PBS, and then incubated with the secondary antibody for 1 h at room temperature. Images of the slides were captured using a WaveFX confocal microscope (Quorum Technologies).

### Statistical Analysis

Statistical analyses were performed using GraphPad Prism version 6.0. All data are presented as mean ± SEM. Normality of each data set was assessed before statistical testing. Parametric tests were applied when the data conformed to a normal distribution; otherwise, appropriate nonparametric tests were used. For experiments with a sample size of 3, nonparametric tests were used to analyze data due to the limited sample size and nonnormal distribution. A *P* value of <0.05 was considered statistically significant.

## RESULTS

### Alteration of VWF mRNA Levels and Protein in Lungs Maintained Under Cold Storage

**FIGURE 1.**
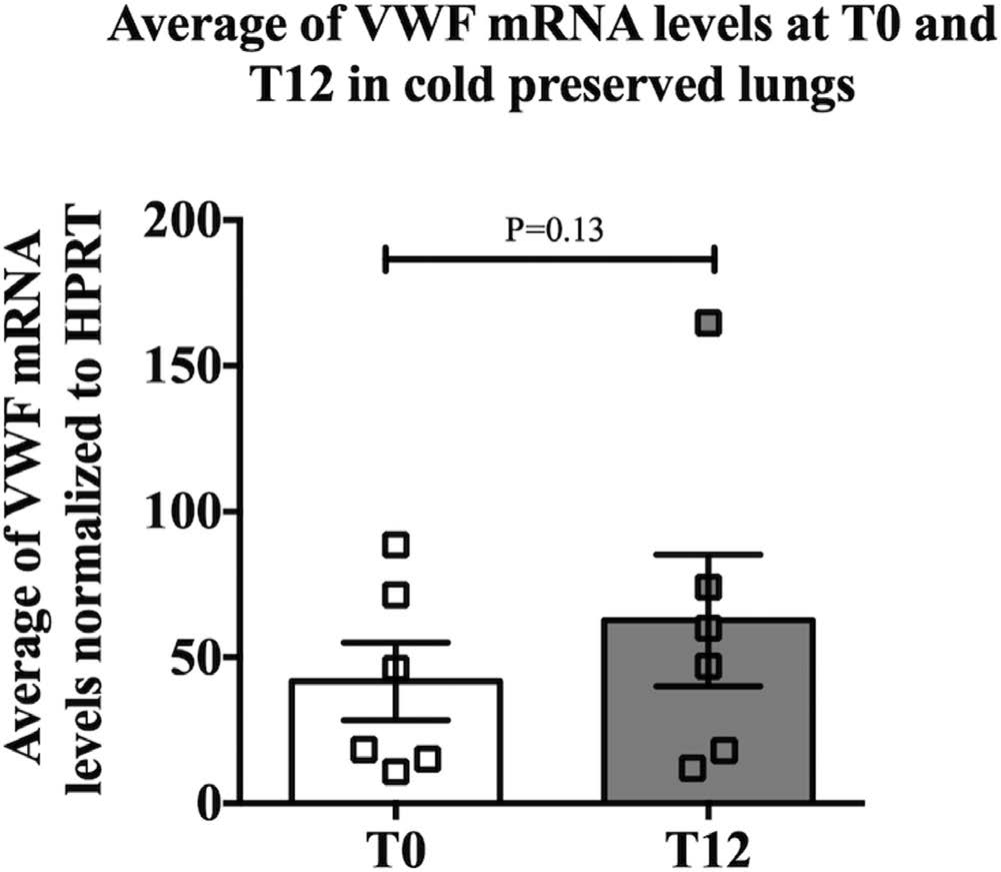
Analysis of VWF mRNA levels at T0 and T12 from SCS-preserved lungs. Quantitative real-time PCR analysis was used to determine the mRNA levels of VWF, normalized to HPRT mRNA, in lung samples at T0 (white bar) and after 12 h in SCS (T12) (gray bar). Average levels of VWF mRNA were reported for the 6 independent experiments (n = 6 per group). Data are shown as the mean ± SEM. (*P < 0.05). HPRT, hypoxanthine‑guanine phosphoribosyltransferase; PCR, polymerase chain reaction; SCS, static cold storage; VWF, Von Willebrand factor.

To determine whether standard cold preservation procedures for organs influence the level of VWF expression, we used porcine lungs procured and maintained under SCS for 0 or 12 h. VWF mRNA levels were measured using quantitative RT-PCR from 6 independent samples at both time points. The results demonstrated that there was no significant increase in VWF mRNA levels (normalized to hypoxanthine‑guanine phosphoribosyltransferase) during a 12-h period (*P* = 0.13; Figure 1; **Figure S1**, **SDC,** https://links.lww.com/TXD/A818).

**FIGURE 2.**
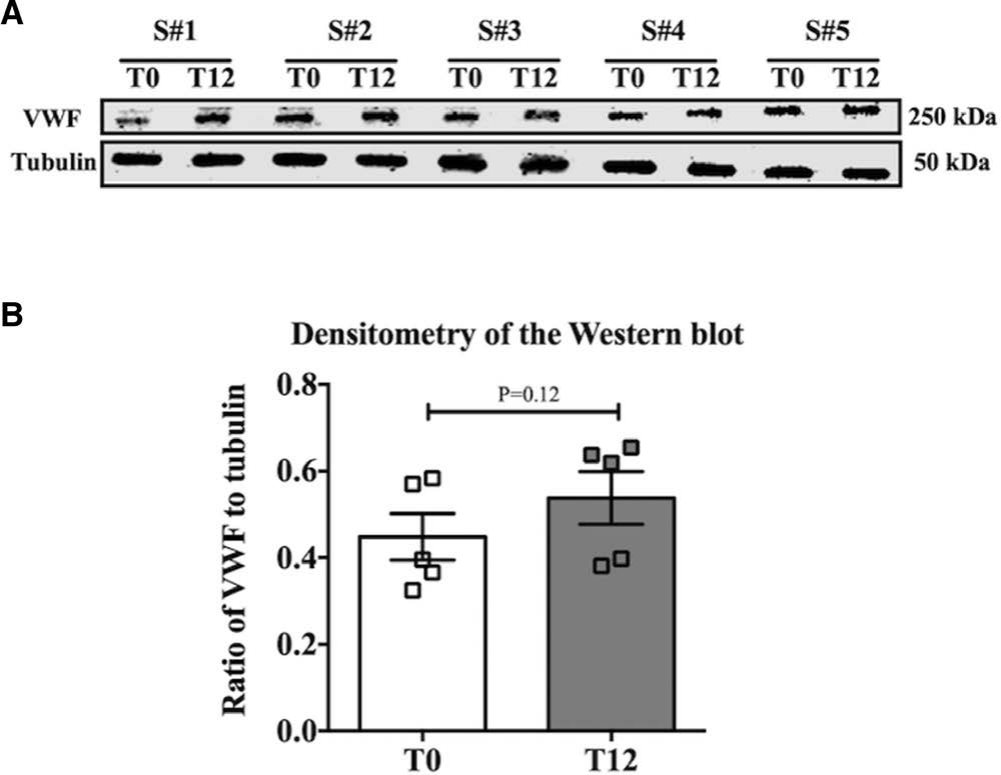
Analysis of VWF protein levels at T0 and T12 from SCS-preserved lungs. A, Western blot analyses were performed to detect VWF protein in preserved lungs at T0 and in the SCS condition for 12 h (T12). Tubulin was used as a normalization control. B, The graphs represent densitometry quantification after normalization to tubulin, averaged from 5 independent animals (n = 5 per group). Data are shown as the mean ± SEM. (*P < 0.05). SCS, static cold storage; VWF, Von Willebrand factor.

Next, to determine whether unaltered VWF mRNA levels were translationally reflected in protein levels, Western blot analyses were performed on 5 pairs of samples used for RNA analysis. The results demonstrated no significant difference in VWF protein levels (normalized to tubulin) after 12 h of SCS (*P* = 0.12; Figure 2; **Figure S2, SDC,** https://links.lww.com/TXD/A818). Cold temperatures suppress the activities of cellular machinery, including transcription and translation. We hypothesize that cold-induced suppression counteracts the induction caused by hypoxia, resulting in similar levels of VWF mRNA and protein at T12 and T0.

### VWF Expression Pattern in SCS-preserved Lungs Pre- and Posttransplantation

Our previous study of hypoxia-induced alterations in VWF expression in mouse lungs demonstrated a significant increase in the proportion of lung microvascular ECs that exhibited de novo activation of VWF expression.^19^ To determine whether SCS preservation similarly affects VWF expression pattern in porcine lung vascular ECs, double-stained immunofluorescence analyses using CD31 (EC marker) and VWF antibodies were performed on lungs preserved under SCS and then transplanted. Samples included IV, PT0, and PT4 lungs.

**FIGURE 3.**
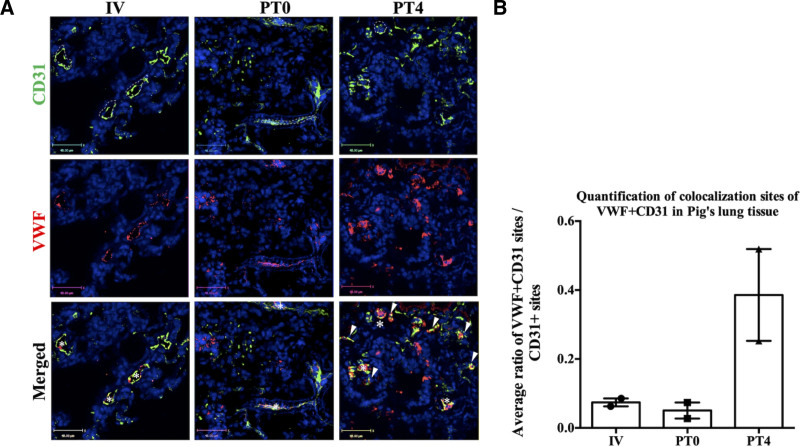
Immunofluorescent analysis of lungs that were kept in SCS before and after transplant. A, Immunofluorescence analysis was performed using double staining for CD31 (green) and VWF (red) on porcine lung samples as described in the Materials and Methods section, including IV, PT0, and PT4.* Represents colocalization of VWF and CD31 in a large/medium vessel. Arrows indicate colocalization of VWF and CD31 in small vessels. Representative large/medium vessels are marked with dotted lines in the image. Scale bar is 48 μm. B, Quantification analysis was performed to identify the ratio of VWF expressing CD31-positive vessels to total CD31-positive vessels based on analyses of 4–5 fields of view of lung sections from 2 pigs for each group (n = 2). Data are shown as the mean ± SEM. SCS, static cold storage; VWF, Von Willebrand factor.

The data revealed a significant increase in ECs (as indicated by CD31 staining) in small vessels that expressed VWF at PT4 compared with IV and PT0 (Figure 3A). Quantitative analysis, presented as the ratio of VWF and CD31 positive sites (indicative of ECs expressing VWF) to total CD31 positive sites (representing total ECs) per field of view, demonstrated that SCS preservation before transplantation alters the VWF expression pattern, leading to an increased number of microvascular ECs expressing VWF (Figure 3B). Due to the extremely limited sample size (n = 2 per group), statistical analysis was not feasible. However, the observed differences between groups were substantial and consistent. These preliminary findings suggest a potentially meaningful biological effect that may achieve statistical significance with a larger sample size.

### Alteration of VWF mRNA Levels and Protein in Lungs Maintained Under Warm Perfusion

It has been reported that using PPV in a normothermic EVLP setting, with both acellular and RBC-based perfusate solutions, has improved the rate of transplantable organs procurements.^28-30,33^ In addition, our group previously demonstrated that the utilization of NPV during EVLP, which most closely mimics physiological conditions, can improve outcomes, regardless of perfusate solution composition.^33^ To see whether exposure to warm perfusion (instead of SCS) affects the levels of VWF expression, the samples that were exposed to warm perfusion with various perfusate and ventilation modes were tested for VWF mRNA and protein expression levels.

**FIGURE 4.**
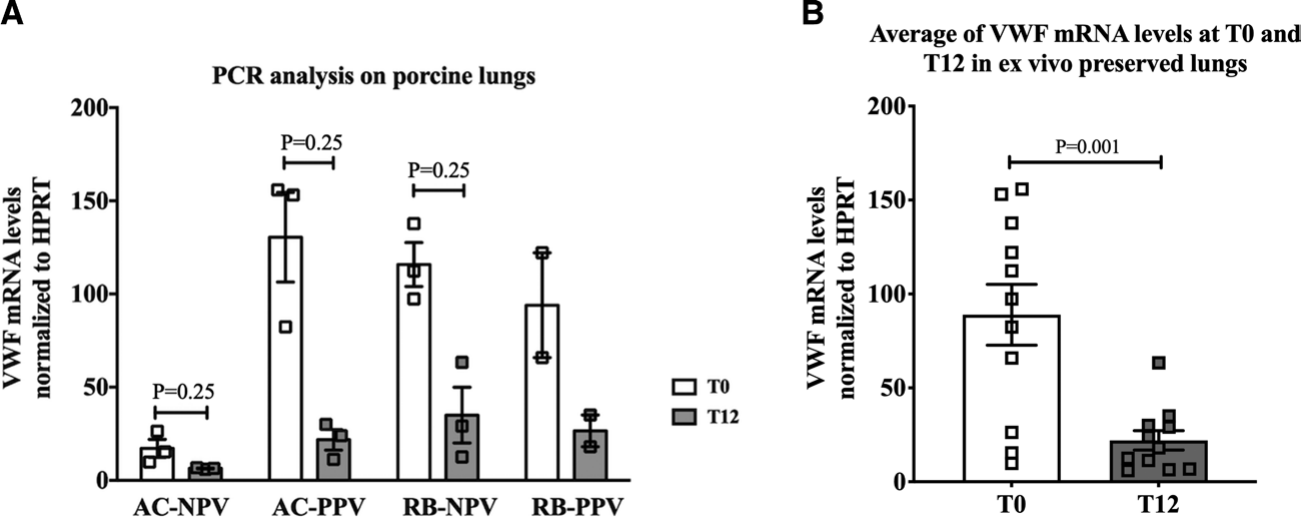
Quantitative real-time PCR analysis of VWF mRNA in EVLP perfused lungs. A, VWF mRNA levels of lungs at T0 and after 12-h EVLP perfusion under various conditions were determined by qRT-PCR. HPRT mRNA was used as a control for normalization. B, Combined analysis of VWF mRNA levels of lungs at T0 and after 12 h EVLP across all perfusate types and ventilation methods. Data are shown as the mean ± SEM. (*P < 0.05). AC-NPV, negative pressure volume with acellular perfusate; AC-PPV, positive pressure volume with AC perfusate; EVLP, ex vivo lung perfusion; HPRT, hypoxanthine‑guanine phosphoribosyltransferase; PCR, polymerase chain reaction; pRBC, packed red blood cell; qRT-PCR, quantitative reverse transcriptase-PCR; SCS, static cold storage; RB-NPV, NPV with pRBC perfusate; RB-PPV, PPV with pRBC perfusate; VWF, Von Willebrand factor.

Tissue samples were collected from procured lungs at T0 and T12 of EVLP as described in the Materials and Methods section. The samples were used for RNA preparation and RT-PCR analyses to detect VWF mRNA as described previously for SCS lung samples. The results demonstrated that VWF mRNA levels were reduced in all of the EVLP conditions at T12 compared with T0, including AC-PPV (*P* = 0.25), RB-NPV (*P* = 0.25), RB-PPV (*P* = 0.25), and AC-NPV (n = 2 unable to do statistical analysis) conditions. Although the data suggest a consistent reduction, they did not achieve statistical significance, likely due to limited sample size (Figure 4A). However, when all data were combined, irrespective of perfusate type and ventilation method, a significant reduction in VWF mRNA expression was observed at T12 compared with T0 (*P* = 0.001; Figure 4B).

**FIGURE 5.**
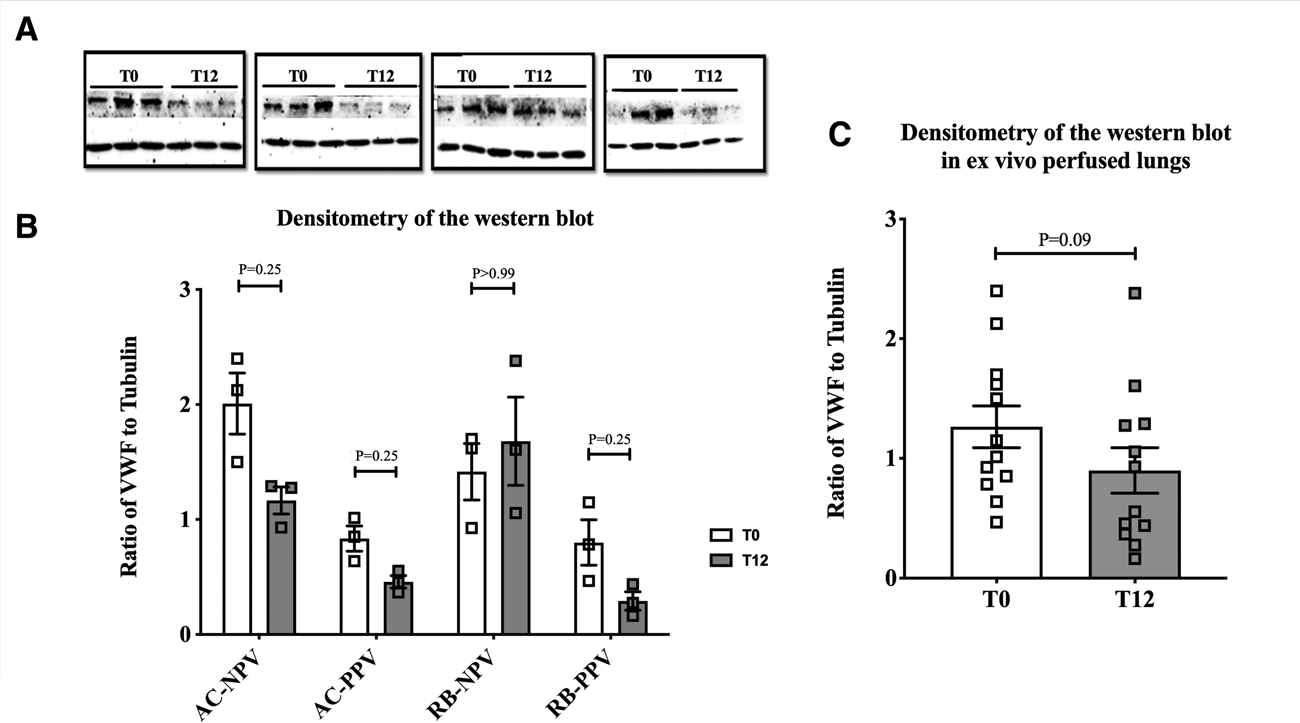
Analysis of VWF protein expression levels in lung samples under EVLP at different conditions. A, Western blot analysis of lung tissues at T0 and after 12-h EVLP perfusion under various conditions. B, The graphs represent quantitative analysis of VWF signals after normalization to the tubulin. Results of 3 independent porcine (n = 3 per group) are shown in all groups. C, Combined analysis of VWF protein levels of lung tissues at T0 and after 12-h EVLP across all perfusate types and ventilation methods. Data are shown as the mean ± SEM. (*P < 0.05). AC-NPV, negative pressure volume with acellular perfusate; AC-PPV, positive pressure volume with AC perfusate; EVLP, ex vivo lung perfusion; pRBC, packed red blood cell; RB-NPV, NPV with pRBC perfusate; RB-PPV, PPV with pRBC perfusate; VWF, Von Willebrand factor.

To determine whether these alterations in VWF mRNA were reflected at the protein level, Western blot analyses were performed on tissue samples from the 4 EVLP-treated groups. The results were generally consistent with the RNA analyses, showing a reduction in VWF protein levels in lungs perfused under EVLP for 12 h compared with the control. Although not statistically significant, the observed reduction may be biologically relevant (Figure 5).

Collectively, these results demonstrate that exposure to warm perfusion and reduced hypoxic and cold exposure time reduces VWF expression in procured lungs.

### VWF Expression Pattern in Lungs That are Maintained Under Warm Perfusion Pre- and Posttransplantation

**FIGURE 6.**
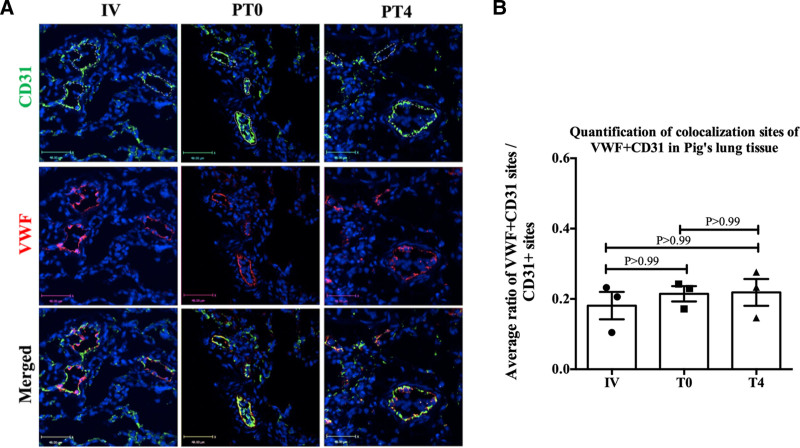
Immunofluorescent analysis of lungs that were kept under EVLP before and after transplant. A, Immunofluorescence analysis was performed using double staining for CD31 (green) and VWF (red) on porcine lung samples as described in the Materials and Methods section, including IV, PT0, and PT4. Representative large/medium vessels are marked with dotted lines in the image. Scale bar is 48 μm. B, Quantification analysis was performed to identify the ratio of VWF expressing CD31-positive vessels to total CD31-positive vessels based on analyses of 5 fields of view of lung sections from 3 pigs for each group (n = 3). Data are shown as the mean ± SEM. (*P < 0.05). EVLP, ex vivo lung perfusion; VWF, Von Willebrand factor.

Because our data demonstrated that SCS preservation altered the VWF expression pattern in porcine lung microvascular ECs, we proceeded to determine whether EVLP also alters the VWF expression pattern in lung ECs. Similar to our approach for the SCS group, we conducted double-stained immunofluorescence analyses targeting CD31 and VWF on lung samples preserved under EVLP both before and after transplantation. Samples included IV, PT0, and PT4 (Figure 6A). Quantitative analysis is presented as the ratio of vessels expressing VWF to the total number of vessels observed per field of view (Figure 6B).

The data demonstrated that there were no significant alterations in VWF expression patterns in the lung vasculature in either PT0 or PT4 compared with IV (*P* > 0.99). The majority of VWF expression was detected in large vessels under all 3 conditions, demonstrating that preservation of lungs under EVLP conditions before transplantation does not alter VWF expression and distribution patterns.

### Blood Vessels of Limb

Because we previously reported that hypoxia regulated VWF expression in an organ-specific manner,^19^ in the current study, we sought to investigate whether the VWF response to the organ preservation condition also exhibits organ specificity. Toward this goal, we used blood vessels isolated from pig’s limb, which were maintained under normothermic perfusion (RBC perfusate at 38 °C) for 0 or 12 h. The results showed a reduction in VWF at both mRNA and protein levels in the samples of the blood vessels of limb at T12 compared with T0 (**Figures S3 and S4, SDC,** https://links.lww.com/TXD/A818). There was a consistent reduction in both mRNA and protein levels at T12 compared with T0. However, the reduction in protein levels was not statistically significant due to the small sample size (*P* = 0.25). These results suggest that organ preservation methods, which reduce vascular exposure to hypoxic and cold conditions, reduce VWF expression levels, and that this VWF response is not restricted to the lung.

## DISCUSSION

Providing the best storage conditions for lungs before transportation is a vital step to minimize one of the major deleterious lung transplant complications, which is microthrombus formation.^7^ VWF is known as a key player molecule in platelet aggregate formation and thrombosis. VWF levels and the pattern of expression in the lung were shown to be altered in response to hypoxia,^19^ which is a condition that is normally experienced by organs preserved for transplantation.

In our study, we used 2 models of organ preservation before transplantation to investigate the effects of these pretransplantation conditions on VWF expression. These models include the standard method of lung preservation, SCS, and the novel EVLP, which reduces the duration of hypoxic and cold exposure.

Our analysis demonstrated that after 12 h in the SCS condition, VWF mRNA and protein levels in lung samples showed no significant change in levels compared to the T0 control groups (Figures 1 and 2). Generally, under cold conditions, many cellular processes, including transcription, translation, and metabolic activities, are significantly reduced, resulting in reduced levels of many gene products.^35^ Thus, under SCS conditions, a balance between hypoxia-induced upregulation and cold-induced downregulation of VWF may lead to similar mRNA and protein expression levels at T12 and T0.

Furthermore, we tested the effect of preservation conditions on the distribution pattern of the VWF vascular tree in lungs. Previously, heterogeneity in VWF expression in the porcine lungs has been reported.^36^ ECs from macro vasculature, including PA and veins, strongly express VWF, whereas ECs from micro vessels, such as alveolar capillaries, either do not express VWF or express VWF at very low levels.^37^ However, in our study, we demonstrated that although in pretransplant lungs VWF expressions were barely detectable in a few small vessels, in SCS maintained lungs, the number of microvasculatures that expressed VWF significantly increased (Figure 3). This result is consistent with our previous study, which demonstrated that hypoxia exposure activates de novo VWF expression in the small-vessel ECs of the lung.^19^

Although there were no net changes in VWF mRNA or protein production during SCS, the VWF-upregulating effect of hypoxia appears to emerge at transplantation, when metabolism increases to physiological levels as a result of normothermia and restored perfusion. This may consequently increase the risk of thrombus formation and organ dysfunction in the recipient after transplantation.

In contrast, EVLP provides nutrition and oxygen that circulate through the organs (similar to physiological conditions) outside the body. Regardless of perfusate and ventilation type, the data demonstrated a statistically significant reduction in VWF mRNA and a trend toward reduced protein levels in the EVLP groups (Figures 4 and 5). Although the decrease in protein levels was evident, it did not reach statistical significance. However, it aligns with the overall pattern of reduction observed and may be biologically important.

Previously, our group showed that lung oxygenation during EVLP improved with cellular perfusate but did not alter significantly between 2 different ventilation techniques (PPV or NPV).^33^ Current study suggests that as long as enough oxygen is provided to the organs by EVLP setting, irrespective of ventilation type, this procedure is highly effective in reducing the expression of the procoagulant molecule, VWF.

We observed considerable variability in VWF mRNA and protein levels at T0 between the SCS and EVLP groups, as well as among different EVLP groups; specifically, AC-NPV at T0 was markedly lower than the other 3 groups. We propose that this variability may reflect the timing of T0 sample collection across groups. In the SCS group, T0 samples were collected immediately upon procurement and placed in the cooler, resulting in fast and minimal processing and likely representing steady-state basal VWF levels. However, in the EVLP groups, T0 samples were obtained after the lungs were procured, transferred, and attached to the EVLP device, but before the initiation of ventilation or perfusion, a process that generally took 15–20 min. Thus, T0 for the SCS group represents immediate postprocurement samples, whereas T0 for the EVLP groups includes a short period of warm ischemia and hypoxic conditions, which we expect to upregulate VWF. The reason for this discrepancy is to enhance the external validity of our methodology, as in clinical practice, the act of attaching lungs to EVLP requires more time than the gold standard of submerging SCS lungs in Perfadex within 3 layers of plastic bagging before placing them on ice. In this way, our methods exactly replicated the comparison of SCS and portable EVLP. Our group previously reported that the initiation of EVLP (T0) (irrespective of perfusate and ventilation strategy) elicits a rapid upregulation of protective genes and inflammatory/stress-mediated genes associated with acute lung injury, compared with SCS,^38^ which is consistent with a potential effect on VWF gene expression.These differences in sample handling may contribute to the observed variations in VWF levels between the T0 samples of EVLP compared with SCS, as well as between AC-NPV and the other 3 EVLP groups. This finding underscores the importance of sample handling and timing as these factors can significantly influence VWF expression outcomes. Despite the variability in T0 VWF levels, we found that 12 h of EVLP significantly downregulated VWF, highlighting the potential of this procedure to reduce the risk of future thrombotic complications in transplanted organs.

Another potential factor contributing to the differences observed in T0 among the EVLP groups may be genetic variation across different cohorts of porcine used for these analyses. Although we used animals from a similar genetic pool, individual variability in VWF levels can be significant, as observed in humans and demonstrated by the T0 VWF levels among the 6 animals used for SCS in Figure 1. The specific cohort of 3 pigs used for AC-NPV may have had inherently lower basal levels of VWF. To account for potential variability that may affect the interpretation of results from small sample sizes, we designed our experimental approach to allow for comparisons of VWF levels at T12 to their corresponding T0 levels within samples from the same animal.

Immunofluorescence analysis demonstrated that transplanted lungs maintained under EVLP exhibited VWF expression mainly in the ECs of large/medium vessels, resembling an IV (pretransplant) sample (Figure 6). This result suggests that EVLP not only reduces the level of VWF expression in the lungs but also preserves the distribution pattern of VWF in large vessels, a feature not observed in SCS-preserved transplanted lungs.

Previously, we reported that ECs from different organs exhibit heterogeneity in their response to hypoxia with regard to VWF expression.^19^ Thus, we proceeded to determine whether the EVLP method that dampens hypoxia-induced VWF upregulation is also observed in other organs, in addition to lung. Limbs from porcine that were similarly maintained under ex vivo perfusion exhibited a similar response to VWF (**Figures S3 and S4, SDC,** https://links.lww.com/TXD/A818), demonstrating that the effect is not restricted to the lung and may be applicable to multiple organs.

Reduction of VWF expression through ex vivo normothermic perfusion may have a significant effect in reducing potential thrombogenic complications. However, it should be noted that thrombus formation is a complex process that includes other factors in addition to VWF. For instance, fibrin deposition and/or catalytic molecules, including VWF-cleaving enzymes, could be altered in response to hypoxia and consequently affect the process of thrombus formation. Nevertheless, modulation of VWF response, as a primary initiator of thrombosis, may be highly effective in reducing thrombotic complications.

Although our current study provides valuable insights into VWF expression under various conditions, we acknowledge that further analysis could enhance our understanding of the functional significance of VWF localization. Future studies could focus on differentiating and examining surface and intracellular VWF to clarify their roles in posttransplant organs. This approach could provide a deeper understanding of the mechanisms driving VWF expression and activity, particularly in response to hypoxic conditions.

Shear stress is a critical variable that significantly influences VWF expression and dimer/multimer formation.^39^ Future studies could also investigate the combination of this variable with hypoxia on organ preservation and posttransplant effects in more detail.

### Limitation

Although our study is limited by a small sample size, the findings suggest that the EVLP technique holds promise in attenuating posttransplant thrombotic risk, thereby potentially improving graft viability and preserving long-term vascular integrity in transplanted organs. Nonetheless, these preliminary results warrant validation in future studies involving larger, more homogenous cohorts.

In addition, this research was conducted using a porcine model, which, although valuable for its physiological similarities to human lungs, may not fully replicate human responses. Therefore, further studies involving human lung assessments are necessary to validate and extend these findings.

## Supplementary Material


