# Supplementary material for: Association of Organ Preservation Methods With Von Willebrand Factor Upregulation in Microvascular Endothelial Cells Posttransplantation: Ex Vivo Lung Perfusion and Static Cold Storage
Source: Transplant Direct. 2026 Jan 12;12(2):e1898. doi: 10.1097/TXD.0000000000001898 (PMC12795047; doi:10.1097/TXD.0000000000001898)
Supplement: Supplementary file 1 [file txd-12-e1898-s001.pdf]

Supplementary:

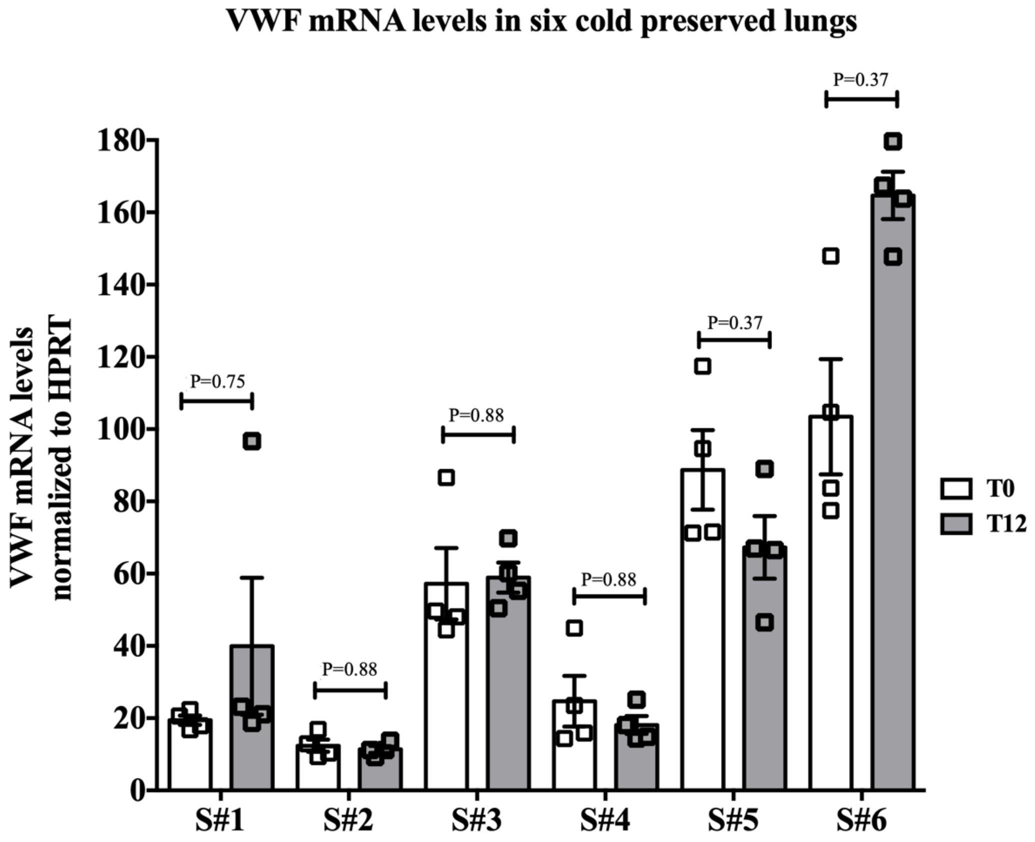

Figure S1. **Analysis of VWF mRNA levels at T0 and T12 from SCS preserved lungs six individual porcine.** Quantitative real time PCR analysis was used to determine the mRNA levels of VWF, normalized to HPRT mRNA, in lungs samples at T0 (white bar) and after 12 hours in SCS (T12) (black bar). Results of 6 independent experiments are shown individually. Error bars are representation of four technical replicates for each sample. (S#1= Sample 1, S#2= Sample 2,

S#3= Sample 3, S#4= Sample 4, S#5= Sample 5 and S#6= Sample 6). Data are shown as the mean ± SEM.

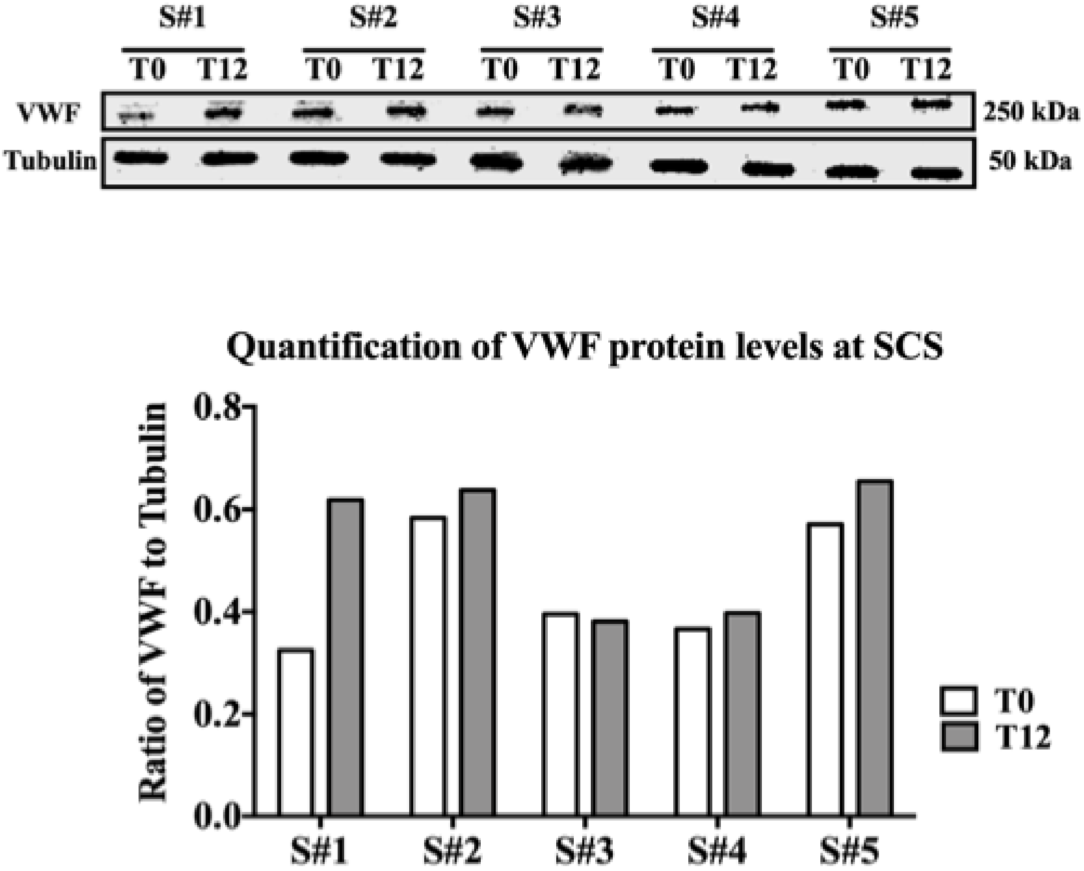

Figure S2. Immunoblotting quantification to compare VWF protein levels at T0 and T12 in each individual pair (T0 and T12) of preserved lung samples in SCS. Western blot analyses were performed to detect VWF protein in preserved lungs at T0 and in SCS condition for 12 hours (T12). The graphs represent densitometry quantification after normalization to tubulin in each individual animal. (S#1= Sample 1, S#2= Sample 2, S#3= Sample 3, S#4= Sample 4, S#5= Sample 5).

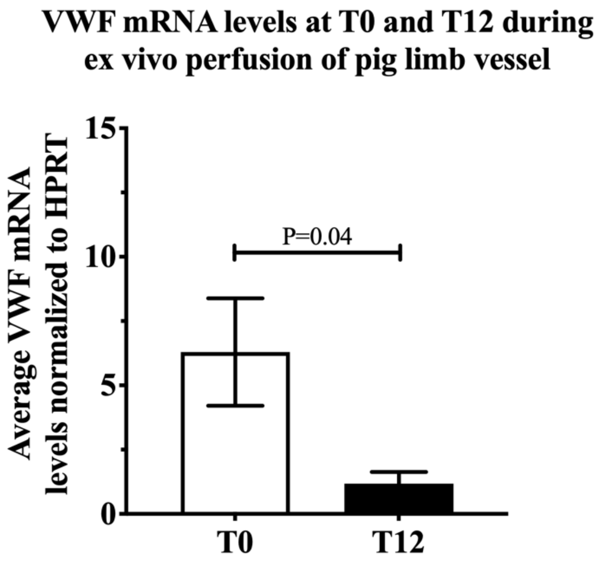

Figure S3. VWF mRNA detection at T0 and T12 samples from normothermic perfused limbs. Quantitative real time PCR analysis was done to determine the mRNA levels of VWF, normalized to HPRT, in vessels of limbs at T0 (white bar) and after 12 hours perfusion (T12) (black bar). Average level of VWF mRNA were reported in the graph. Data are shown as the mean ± SEM.

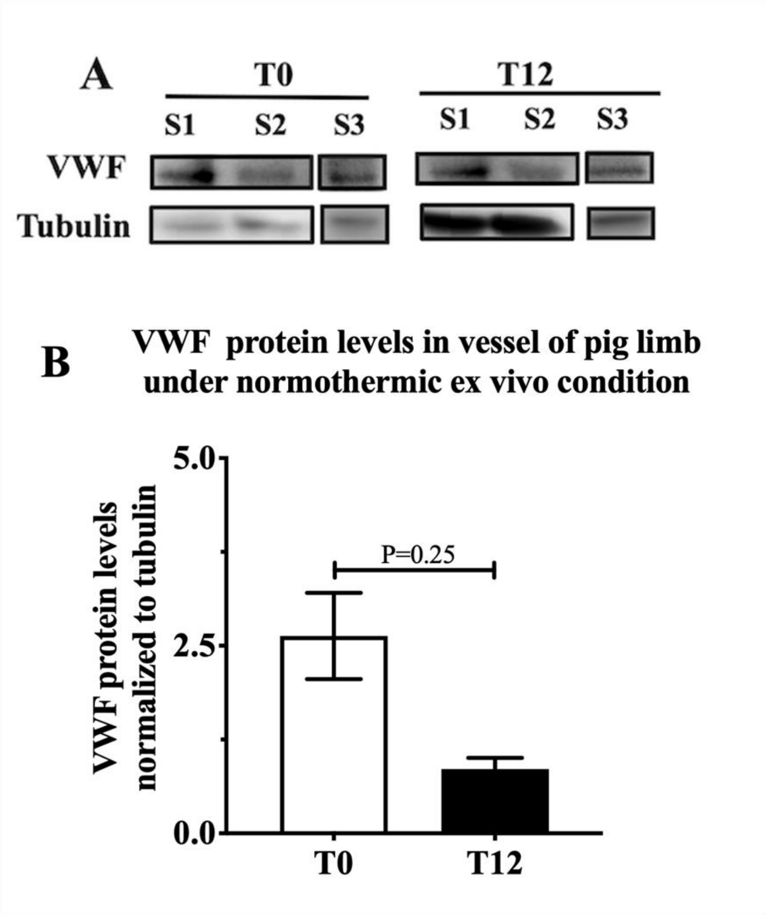

Figure S4. Immunoblotting analysis to detect VWF protein levels at T0 and T12 vessels samples from perfused limbs. (A) Western blot analyses to detect VWF protein in ex vivo perfused limb's vessels at T0 and T12 time points. Tubulin was used as the loading control. **(B)** Immunoblotting quantification is reported as a graph after normalization to tubulin from 3 independent experiments (n=3). (S1= Sample#1, S2= Sample #2, S3= Sample #3). Data are shown as the mean ± SEM.
